# Potential treatment of glutathione in bullfrogs with abnormal hepatic lipid metabolism revealed by hepatic lipid metabolism and serum metabolomics analysis

**DOI:** 10.3389/fcimb.2024.1426340

**Published:** 2024-08-13

**Authors:** Zehui Su, Fu Gao, Rui Shu, Kai Cai, Shugaung Fang, Xiaoting Lei, Dan Li, Kun Hu

**Affiliations:** ^1^ National Demonstration Center for Experimental Fisheries Science Education (Shanghai Ocean University), Shanghai, China; ^2^ National Pathogen Collection Center for Aquatic Animals, Shanghai, China; ^3^ Key Laboratory of Freshwater Aquatic Genetic Resources, Ministry of Agriculture, Shanghai, China; ^4^ Henan ANIMIC Biotechnology Co., Ltd., China; ^5^ Guangdong Xingwa Agricultural Technology Co., Ltd., Zhaoqing, China; ^6^ Wecare Probiotics Co., Ltd, China; ^7^ Shanghai Police College, Shanghai, China

**Keywords:** glutathione, bullfrog, lipid metabolism, plasma, liver disease

## Abstract

**Introduction:**

With the continuous growth of bullfrog supply, it has become an important aquaculture species. Due to the lack of actionable industry standards and regulation, the misuse of anti-disease drugs and abnormal liver lipid metabolism in bullfrogs have become a major obstacle to the development of bullfrog aquaculture industry. Glutathione is a natural tripeptide that can be synthesized intracellularly, and its physiological functions mainly include the treatment of liver diseases, antioxidant, detoxification, anti-tumor, enhancement of immunity, and delaying aging.

**Methods:**

In this study, the therapeutic effect of glutathione on bullfrogs with abnormal liver lipid metabolism was revealed from hepatic lipid metabolism and serum metabolomics analysis. The survival rate, liver histomorphology, serum antioxidant enzyme activity, liver lipase activity and serum metabolomics, liver metabolomics were studied and analyzed by feeding the bullfrogs with abnormal lipid metabolism with glutathione for 20 days in the NC, FI and GSH groups.

**Results:**

The results of the study showed that glutathione was able to repair the liver and improve the survival rate of bullfrogs with abnormal lipid metabolism; the activity of serum SOD enzymes was significantly increased; the activities of ACP and AKP were significantly decreased; the activities of HDL-C and T-CHO were significantly increased; and the activities of LDL-C, TBA, and TG were significantly decreased in the liver; the contents of metabolites, such as PC, PS, and PE were significantly up-regulated, and the levels of up-regulated Autophagy - other, Necroptosis and ErbB signaling pathway, and down-regulated Sphingolipid metabolism, D-Amino acid metabolism metabolic pathway, to some extent The metabolic pathways of Sphingolipid metabolism and D-Amino acid metabolism were down-regulated to alleviate the disorders of glycerophospholipid and amino acid metabolism to a certain extent, thus alleviating the abnormalities of liver lipid metabolism.

**Discussion:**

The results showed that glutathione could effectively treat the liver lipid metabolism disorder of bullfrogs, promote the growth and development of bullfrogs, repair the liver function, reduce the inflammation, and promote the healthy and green development of bullfrog industry.

## 1 Introduction

Bullfrog belongs to Amphibia, Frog family, native to North America, is an aquaculture animal with high efficiency in producing high quality protein, which was introduced to our country from Cuba in the 1960s. Because of its short breeding cycle, water-saving and energy-saving, low investment, high yield, low technology content and other characteristics, bullfrog farming is prevalent in coastal areas of China, such as Fujian, Guangdong, Zhejiang, etc. Meanwhile, food and beverage enterprises gradually began to use bullfrog products. At the same time, various catering enterprises gradually began to use bullfrog products, forming a good situation of production and marketing, and many large-scale bullfrog farms have appeared in the country since 2010. With the development of domestic bullfrog catering and demand, superimposed on policy assistance, China’s bullfrog supply continues to grow, and become an important aquaculture species. 2022, the national bullfrog whole industry chain scale of nearly 100 billion yuan.

However, due to the lack of operable industry standards and regulation, the abuse of anti-disease drugs (Casali et al., 2005), serious pollution of aquaculture water and environmental degradation, it leads to overloading of the liver, intestines and other digestive organs of bullfrogs and abnormal lipid metabolism, which results in excessive accumulation of liver and abdominal fat, damaging the liver, gallbladder and intestinal organs, and causing diseases such as liver enlargement, hepatitis, enteritis, etc (Keum Taek et al., 2002), which seriously restricts growth and development of bullfrogs and even kills them in serious cases (Wei et al., 2023). Among them, abnormalities of hepatic lipid metabolism in bullfrogs have become a major obstacle to the development of bullfrog farming, causing great losses.

Glutathione is a tripeptide containing a y-amide bond and a sulfhydryl group, and consists of glutamic acid, cysteine and glycine, with the sulfhydryl group on cysteine as its active group. Glutathione has two main forms: reduced glutathione and oxidized glutathione. The ratio of reduced to oxidized glutathione (GSH/GSSG) is often used as a factor to assess cellular antioxidant capacity or cytotoxicity (Hamre et al., 2024). Reduced glutathione is the main state of existence, accounting for about 95% of the total, and is involved in glucose metabolism and the tricarboxylic acid cycle in the body. It can activate a variety of enzymes, such as sulfhydryl (-SH) enzymes - coenzymes, and thus affects the metabolism of sugars, proteins, and fats in the body (Alschuler, 2011). Glutathione can scavenge oxygen free radicals, antioxidant, detoxification and enhancement of immunity (Tsugawa et al., 2019).Glutathione has certain effect on the treatment of liver disease. The abnormalities of liver lipid metabolism in bullfrogs are mainly due to drug liver injury caused by overdosing and indiscriminate use of drugs. The main mechanism of drug induced liver injury is due to the toxicity of the drug itself and the immune reaction, most of the drugs are transformed by hepatocytes, and normal medication will not cause liver injury, but after overdose, a large number of drugs are transformed into water-soluble metabolites by hepatocytes, and the generation of these substances will greatly consume the glutathione in the hepatocytes, resulting in hepatocellular necrosis, and the exogenous glutathione can reduce the toxicity of the drug and the immune reaction, which will cause the liver cell necrosis. Supplementation of exogenous glutathione can reduce the toxic reaction of the drug as well as the immune response, thus playing a role in protecting hepatocytes. In addition, glutathione is also involved in many cellular metabolic activities (Semenova et al., 2022). Studies have shown that an imbalance of glutathione metabolism in the body will directly affect the health of the body, such as Alzheimer’s disease, Parkinson’s disease, cancer (Dirican et al., 2024), chronic liver disease and so on are related to it (Cosar et al., 2024). Studies have shown that exogenous glutathione can be extracted from yeast and plants by solvent extraction, fermentation, chemical synthesis and enzyme synthesis, and it can be used to treat and prevent liver and kidney injury as well as for the recovery of cancer patients after chemotherapy (Cai et al., 2024a). At the same time, glutathione has an inhibitory effect on a variety of viruses.

Although bullfrogs have been cultured in captivity for a long time, there is a gap in the study of bullfrog diseases, especially the study of hepatic lipid metabolism part. Therefore, on the basis of previous studies, this study investigated the therapeutic effects of glutathione on bullfrogs with abnormal hepatic lipid metabolism from the perspective of hepatic lipid metabolism and serum metabolomics, which will provide a reference for future in-depth research on the mechanism of glutathione’s action. We hope to provide technical support for the development of glutathione-related products and their rational application in bullfrog green and healthy aquaculture production, so as to reduce the abuse of antibiotics and contribute to food safety and environmental safety.

## 2 Materials and methods

### 2.1 Test materials

Glutathione was provided by Angel Yeast Co.,Ltd.

Healthy bullfrogs and bullfrogs suffering from abnormal fat metabolism used in this study were purchased from Guanxing (Zhaoqing) Agricultural Science and Technology Co.

### 2.2 Experimental design and feeding management

The aquaculture experiment was conducted at the Digital Fisheries Farm of Stargazer Agriculture. One hundred healthy bullfrogs and 200 bullfrogs with abnormal fat metabolism were selected to be temporarily reared for 2 weeks before the start of the experiment, during which the bullfrogs were fed basic feed twice a day to acclimatize them to the feed and culture environment. At the end of the temporary rearing period, we stopped feeding for 24 h. We selected 80 healthy bullfrogs with uniform size as the healthy group NC, and 50 bullfrogs with bacterial enteritis as the pathological group FI and the treatment group GSH. The initial weight of the test bullfrogs was (75 ± 5) g. The three experimental groups were cultured for 20 days and recorded. The water level of the tanks was three centimetres, the water temperature was 22°C, and the tanks were fed twice a day, with 0.5 g of glutathione per 100 g of feed for the GSH group, and the tanks were changed and cleaned 30 min before feeding. Observations were made in the morning, afternoon and evening of each day during the culture period to record the food intake, activity and mortality of the bullfrogs.

### 2.3 Sample collection

During the culture period, three bullfrogs were dissected from each group every 5 days to observe their gastrointestinal condition. After 20 days of culture, 14 bullfrogs were taken from each tank separately and executed by double destruction of medulla. The livers of 7 bullfrogs were dissected and some of them were fixed in Bern’s solution for liver morphology analysis. The remaining livers were put into sterilized cryopreservation tubes and quickly stored in liquid nitrogen for liver metabolomics analysis and metabolic enzyme activity determination. The other 7 bullfrogs were injected into 1.5 mL centrifuge tubes with a 1 mL syringe, and then centrifuged at 4°C for 10 min (12000 r/min, 4°C) after 24 h. The serum was separated and stored at -80°C for biochemical and metabolomic analyses.

### 2.4 Analysis of index measurements

#### 2.4.1 Liver histomorphometric analysis

Liver tissues of 0.5 cm3 each were removed from Bourne’s fixative, dehydrated with different concentrations of alcohol using a biological tissue automatic dehydrator (KD-TS3A), paraffin-embedded tissues, trimmed the solidified wax blocks, and sliced with a tissue slicer (model: RM2235 slicer produced by leica, Graaf, Germany) with a thickness of 6 μm; the slices were deparaffinized and stained with hematoxylin a After deparaffinization, the slices were stained with hematoxylin and eosin (H.E.), and the slices were sealed and observed and photographed with an orthogonal fluorescence microscope (Leica DM5500B).

#### 2.4.2 Analysis of blood antioxidant indexes

Preparation of serum: 7 bullfrogs were taken from each of the NC group, FI group and GSH group for blood sampling, placed at room temperature for 4~5 h. After precipitating the serum, the serum was centrifuged at 12 000 r/min for 10 min, and then the serum was taken and stored at -80°C for spare.

The kits were purchased from Nanjing Jianjian Bioengineering Institute, and the activities of alkaline phosphatase ALP, acid phosphatase ACP and superoxide dismutase SOD were determined according to the instructions.

#### 2.4.3 Analysis of liver antioxidant indexes

Seven bullfrogs were taken from each of the NC, FI and GSH groups for liver tissue extraction, and the livers were quickly put into liquid nitrogen after removal, and then stored in a -80°C refrigerator for the detection of liver antioxidant indexes.

The kits were purchased from Nanjing Jianjian Bioengineering Institute, and the methods for the determination of total cholesterol (TC), total bile acids (TBA), high-density lipoproteins (HDL), low-density lipoproteins (LDL), and triglycerides (TG) were in accordance with the instructions.

#### 2.4.4 Serum full-spectrum metabolism-LCMS metabolomics analysis

##### 2.4.4.1 Pre-treatment

(1) Remove the samples stored at -80°C, thaw in an ice-water mixture, and pipette 100 μL of sample into a 1.5 mL EP tube;
(2) Add 300 μL of protein precipitant methanol-acetonitrile (V:V=2:1, containing mixed internal standard, 4 μg/mL), vortex and shake for 1 min;
(3) Ultrasonic extraction in an ice-water bath for 10 min, and leave at -40°C overnight;
(4) Centrifuge for 10 min (12000 rpm, 4°C), aspirate 150 μL of the supernatant with a syringe, filter it using a 0.22 μm organic phase pinhole filter, transfer it to an LC injection vial and store it at -80°C until LC-MS analysis.
(5) Quality control (QC) samples were prepared by mixing equal volumes of extracts from all samples.
(6) Note: All extraction reagents were pre-cooled at -20°C before use.

##### 2.4.4.2 Liquid chromatography-mass spectrometry analysis conditions

The analyzing instrument was a liquid-mass spectrometry system consisting of a Waters ACQUITY UPLC I-Class plus/Thermo QE ultra-high performance liquid tandem high-resolution mass spectrometer.

##### 2.4.4.3 Chromatographic conditions

Chromatographic column: ACQUITY UPLC HSS T3 (100 mm×2.1 mm, 1.8 um); column temperature: 45°C; mobile phase: mobile phase: A-water (containing 0.1% formic acid), B-acetonitrile; flow rate: 0.35 mL/min; injection volume: 3 μL. Finally, the elution gradient is shown in **Table 1**.

**Table 1:** Elution gradients.

Time	A%	B%
0.0	95.0	5.0
2.0	95.0	5.0
4.0	70.0	30.0
8.0	50.0	50.0
10.0	20.0	80.0
14.0	0.0	100.0
15.0	0.0	100.0
15.1	95.0	5.0
16.0	95.0	5.0

##### 2.4.4.4 Mass spectrometry conditions

The mass spectrometry parameters are shown in **Table 2**.

**Table 2:** Mass spectrometry parameters.

Parameters	Positive ion	Negative ion
Spray Voltage (V)	3800	-3000
Capillary Temperature (°C)	320	320
Aux gas heater temperature (°C)	350	350
Sheath Gas Flow Rate (Arb)	35	35
Aux gas flow rate (Arb)	8	8
S-lens RF level	50	50
Mass range (m/z)	70–1050	70–1050
Full ms resolution	70000	70000
MS/MS resolution	17500	17500
NCE/stepped NCE	10, 20, 40	10, 20, 40

#### 2.4.5 Full-spectrum liver metabolism-LCMS metabolomics analysis

##### 2.4.5.1 Pre-treatment

(1) Weigh 30 mg of tissue and add 300 μL of methanol-water (V:V=1:1, containing mixed internal standard, 0.1 mg/mL, methanol configuration);
(2) Add two small steel beads, pre-cool at -20°C for 2 min, and add to a grinder (60 Hz, 2 min);
(3) Add 300 μL of chloroform, vortex for 30 s, ultrasonic extraction for 10 min, leave at -20°C for 20 min, centrifuge for 10 min (12000 rpm). Centrifuge for 10 min (12000 rpm, 4°C), take 200 μL of the lower chloroform layer into a new centrifuge tube;
(4) Add 300 μL of chloroform-methanol (V:V=2:1) into the centrifugal tube with the lower layer of solution, vortex for 30 s, and ultrasonic extraction for 10 min in an ice-water bath;
(5) After standing at -20°C for 20 min, centrifuge for 10 min (12000 rpm, 4°C), take 200 μL of the lower chloroform layer and combine with the previous 200 μL, totaling 400 μL;
(6) Take 200 μL into the LC-MS injection vial and evaporate it dry. The lipid residue in the LC-MS injection vial was redissolved with 300 μL of isopropanol-methanol (V:V=1:1), vortexed for 30 s, and sonicated with ice water for 3 min, then the solution was transferred to a 1.5 mL centrifuge tube, and left to stand at -20°C for 2 h. The solution was then centrifuged for 10 min (12000 rpm, 4°C) and centrifuged for 10 min (12000 rpm, 4°C);
(7) Centrifuge for 10 min (12000 rpm, 4°C), 150 μL of supernatant was loaded into an LC-MS injection vial with a liner tube for LC-MS analysis;
(8) Quality control (QC) samples were prepared by mixing equal volumes of extracts from all samples, and the volume of each QC was the same as that of the sample. Note: All extraction reagents were pre-cooled at -20°C before use.

##### 2.4.5.2 Liquid chromatography-mass spectrometry analysis conditions

The analyzing instrument was a liquid-mass spectrometer system consisting of ACQUITY UPLC I-Class plus ultra-high performance liquid tandem high-resolution mass spectrometer.

##### 2.4.5.3 Chromatographic conditions

Column temperature: 55°C; mobile phase A: acetonitrile: water = 6:4 (v/v, containing 10 mM ammonium acetate); mobile phase B: isopropanol: acetonitrile = 9:1 (v/v, containing 10 mM ammonium acetate); flow rate: 0.26 mL/min; injection volume: 3 μL and the elution gradient is shown in **Table 3**.

**Table 3:** Elution gradients.

Time (min)	Flow[ml/min]	Solvent B(%)
0.0	0.26	32.0
1.5	0.26	32.0
15.5	0.26	85.0
15.6	0.26	97.0
18.0	0.26	97.0
18.1	0.26	32.0
20.0	0.26	32.0

##### 2.4.5.4 Mass spectrometry conditions

Mass spectral parameters are shown in **Table 4**.

**Table 4:** Mass spectrometry parameters.

Parameters	Positive ion	Negative ion
Spray Voltage (V)	3500	-3000
Capillary Temperature (°C)	300	300
Aux gas heater temperature (°C)	350	350
Sheath Gas Flow Rate (Arb)	45	45
Aux gas flow rate (Arb)	10	10
S-lens RF level	50	50
Mass range (m/z)	150–1500	150–1500
Full ms resolution	70000	70000
MS/MS resolution	17500	17500
NCE/stepped NCE	25, 35, 45	25, 35, 45

### 2.5 Data analysis

All the experimental data were statistically analyzed using Excel and SPSS 26.0 software, and one-way ANOVA (one-way ANOVA) with Duncan’s multiple test was used for comparison, and the experimental data were expressed as the mean ± standard error, and the significance level was set at (*P<0.05*).

## 3 Results

### 3.1 Effect of glutathione feeding on the survival rate of bullfrog culture

The survival rate of bullfrogs in the experimental group was improved after 20 days of glutathione feeding to bullfrogs (**Table 5**), the mortality rate of the FI group reached 98.0% in 20 days, and the mortality rate of the GSH group fed with glutathione was 68.6%, which was a 29.4% reduction in the mortality rate.

**Table 5:** Comparison of mortality of bullfrogs in NC, FI and GSH groups after 20 days.

Group	test frog/ind	death toll/ind	mortality/%
NC group	51	4	7.8
FI group	51	50	98.0
GSH group	51	35	68.6

### 3.2 Effect of glutathione feeding on the morphology of bullfrog liver

**Figure 1:**
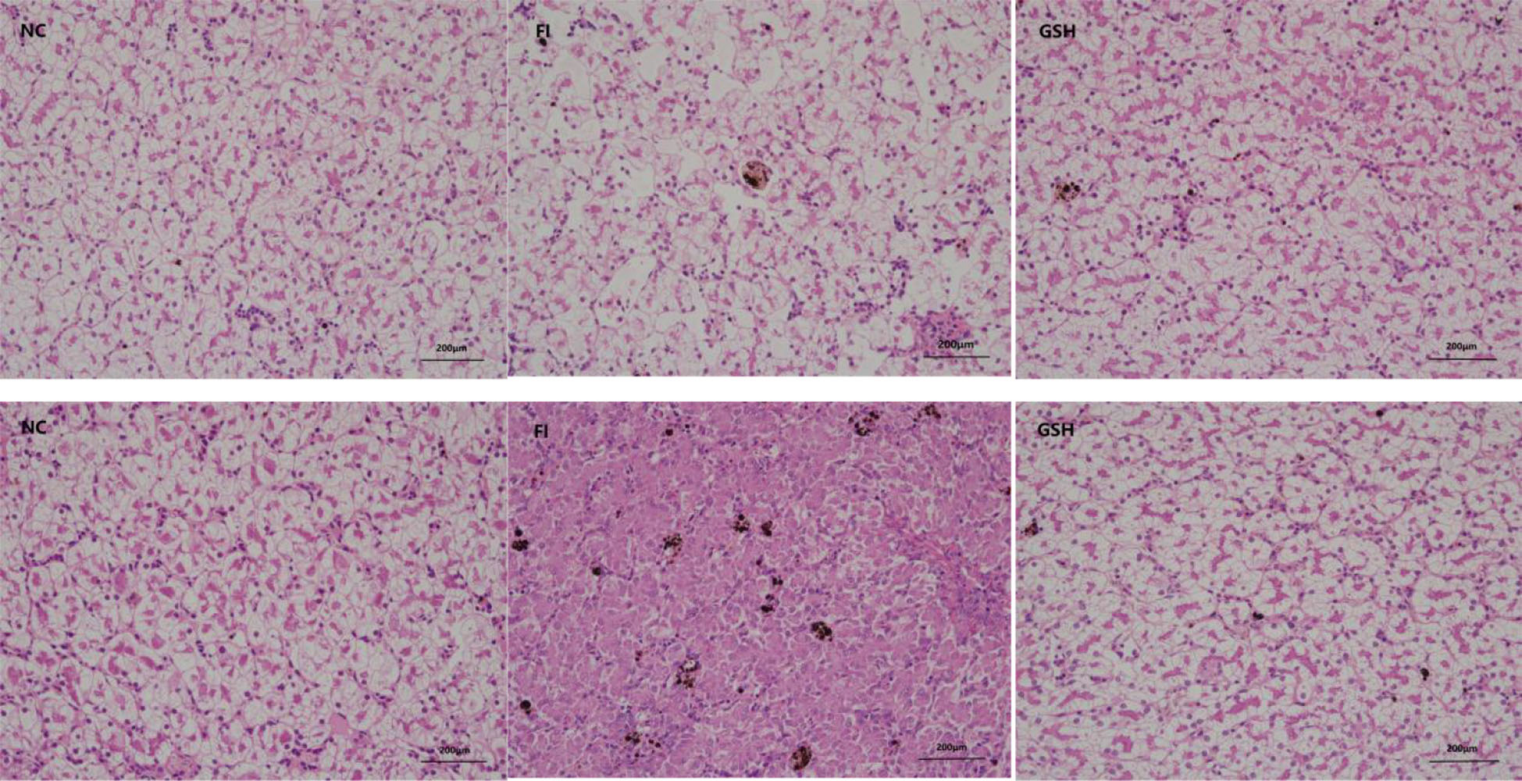
Three sets of bullfrog liver sections. From left to right, NC, FI and GSH groups.

The results of liver tissue sections in the experiment are shown in **Figure 1**, which shows that glutathione has some restorative and therapeutic effects on the liver of bullfrogs.

### 3.3 Effects of glutathione feeding on the activities of immunity-related enzymes in bullfrog serum

**Figure 2:**
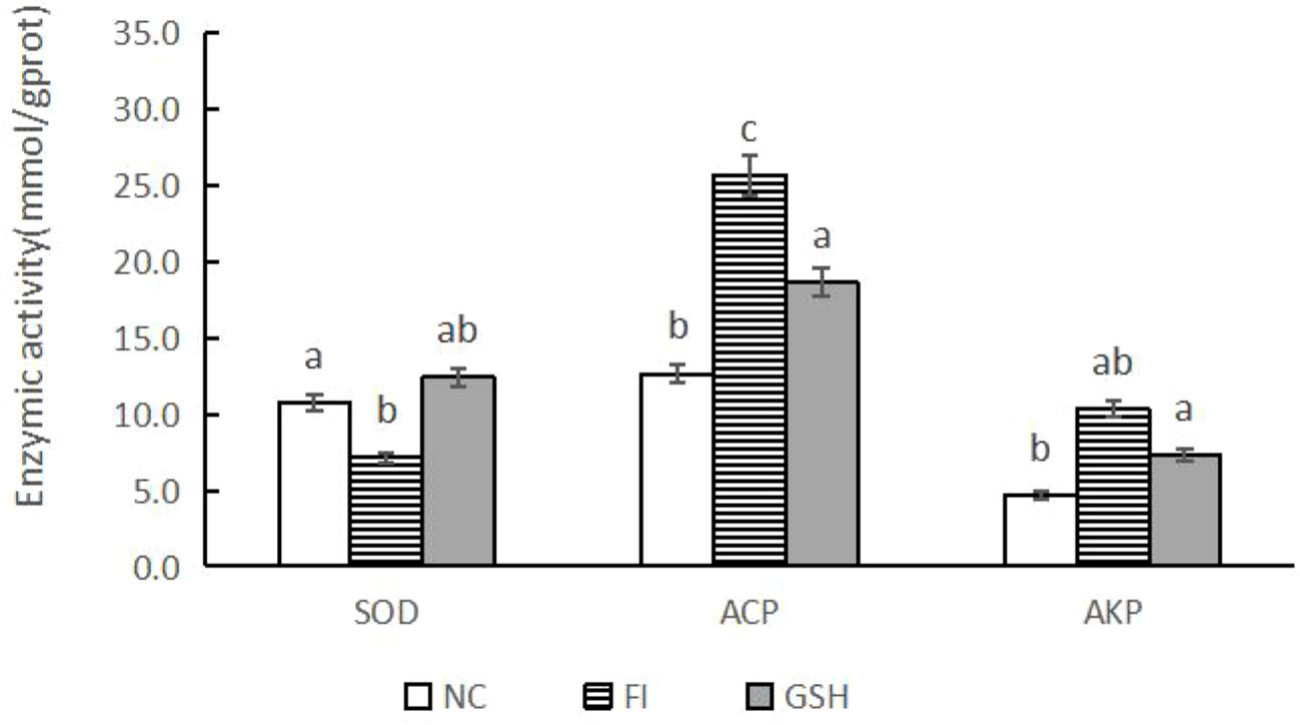
Serum immunometabolizing enzyme activities of bullfrogs in NC, FI and GSH groups. Different lowercase letters labeled on the columns indicate significant differences (*P* < 0.05).

As shown in **Figure 2**, SOD activity was elevated and AKP activity was decreased in the serum of bullfrogs in the glutathione group compared to the FI group. Among them, ACP activity was significantly lower than that in the FI group (*P* < 0.05).

### 3.4 2.4Effects of glutathione feeding on the activities of related metabolic enzymes in bullfrog liver

**Figure 3:**
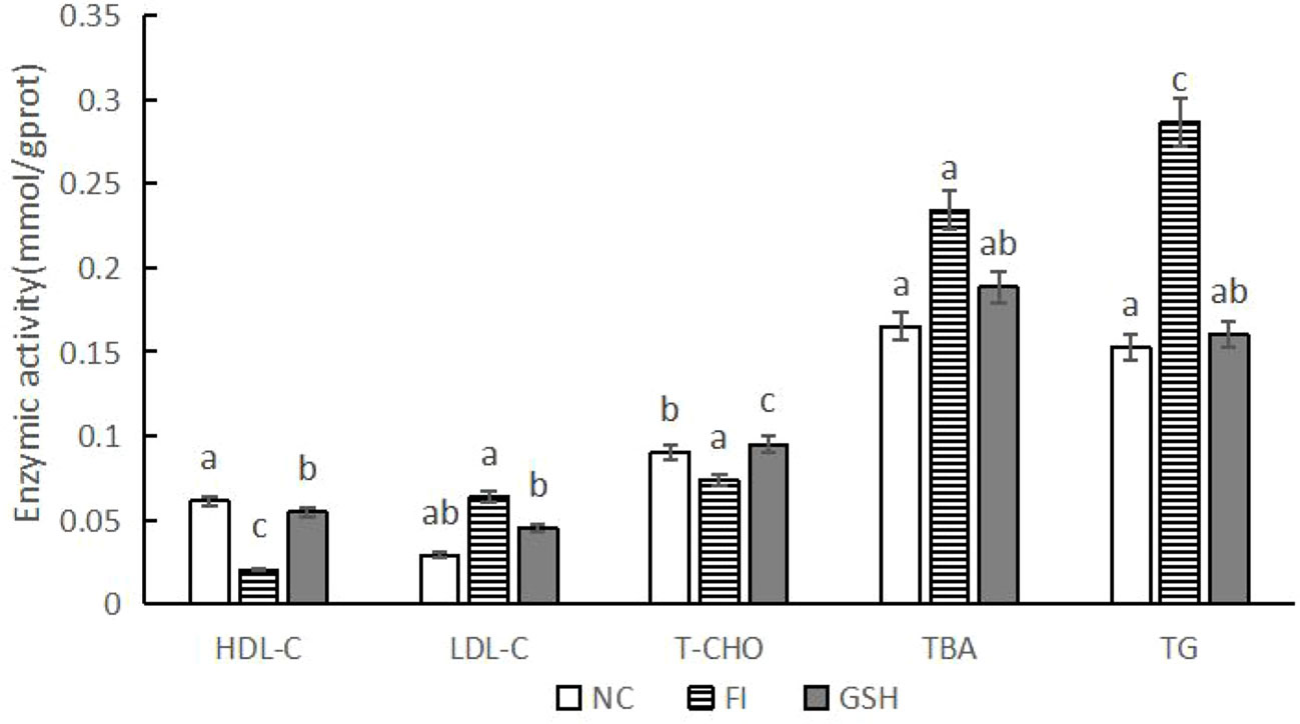
Hepatic lipid metabolizing enzyme activities of bullfrogs in NC, FI and GSH groups. Different lowercase letters labeled on the columns indicate significant differences (*P* < 0.05).

As shown in **Figure 3**, compared with the FI group, the activities of HDL-C and T-CHO were significantly higher in the liver of bullfrogs fed with glutathione; the activities of LDL-C, TBA and TG were significantly lower (*P* < 0.05).

### 3.5 Modulation of serum metabolites in bullfrogs by glutathione mix feeding

#### 3.5.1 Sample raw data and principal component analysis

**Figure 4:**
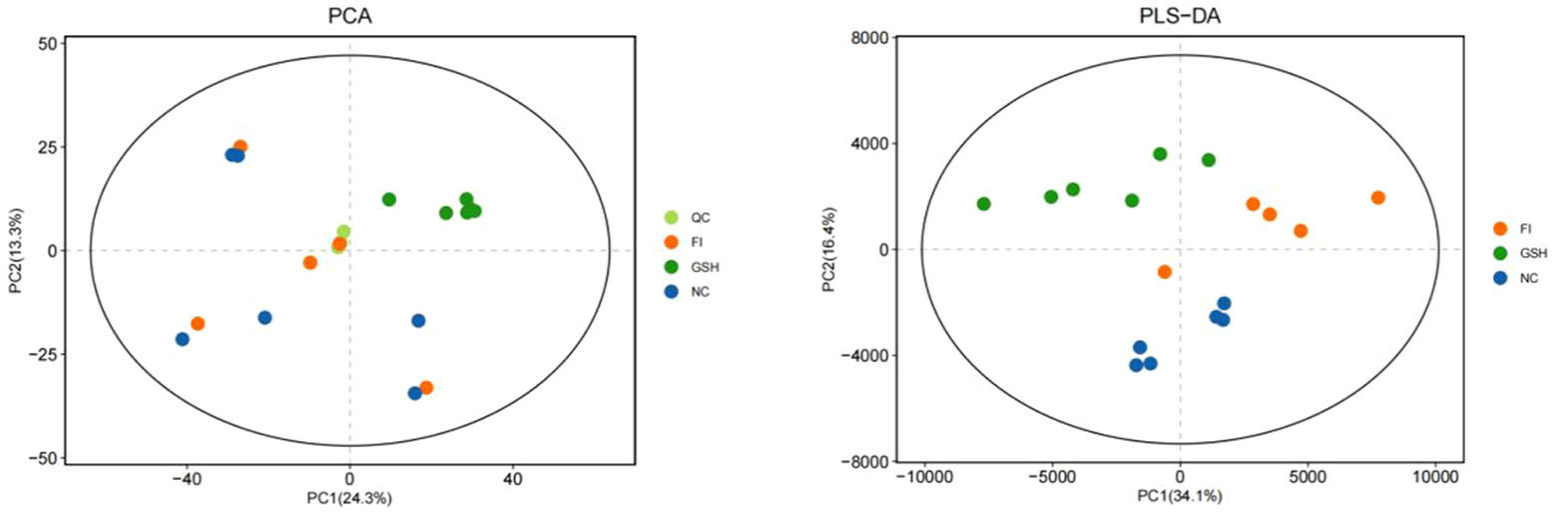
PCA versus PLS-DA plots of serum metabolites in the NC, FI, and GSH groups.

PCA is an unsupervised analysis that responds to the raw data and helps to view the data as a whole. Partial Least Squares-Discriminant Analysis (PLS-DA) is a supervised discriminant statistical method that uses partial least squares regression to model the relationship between metabolite expression and sample grouping to achieve prediction of the sample category. The addition of grouping variables in PLS-DA can make up for the shortcomings of the PCA method, and the parameter R2X (cum) can be used to judge the validity of the model.PLS-DA In addition to the parameter R2X (cum), it also includes the explanation rate R2Y (cum) and the prediction rate Q2 (cum), and the closer the two are to 1, it means that the PLS-DA model can better explain and predict the differences between the two groups of samples, which represents the better the model prediction ability. As can be seen from **Figures 4**, the samples of NC, FI and GSH groups are all in the 95% confidence interval, and can be spatially separated uniformly without overlap, indicating that there are significant differences in metabolites between the two groups, and that they are able to be analyzed in the next step.

#### 3.5.2 Screening of differential metabolites

**Figure 5:**
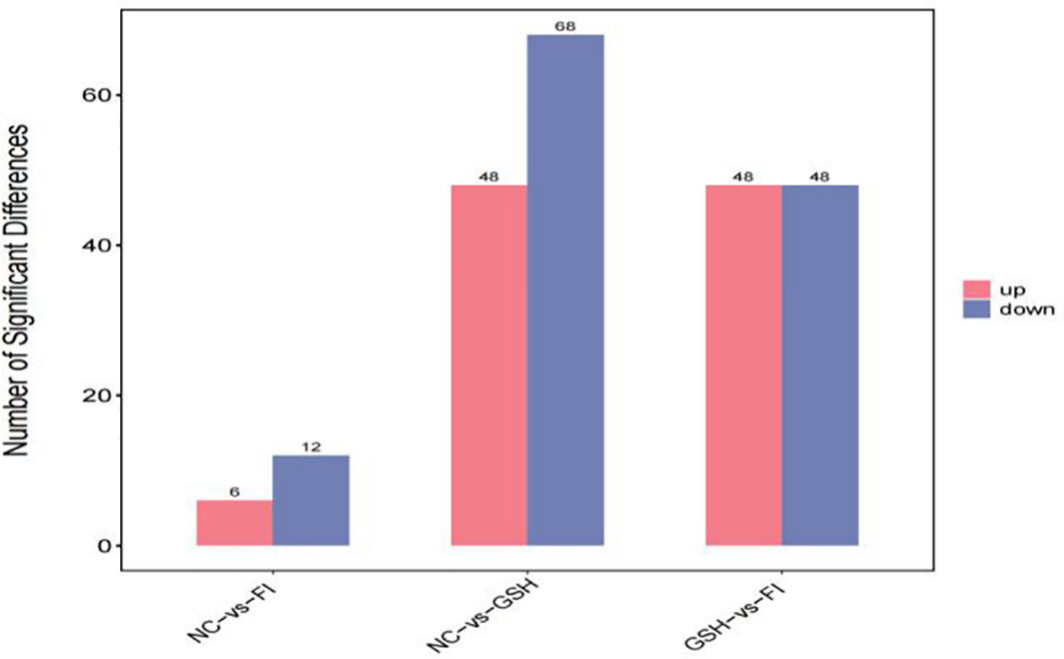
Differential metabolite quantity statistical chart.

**Figure 6:**
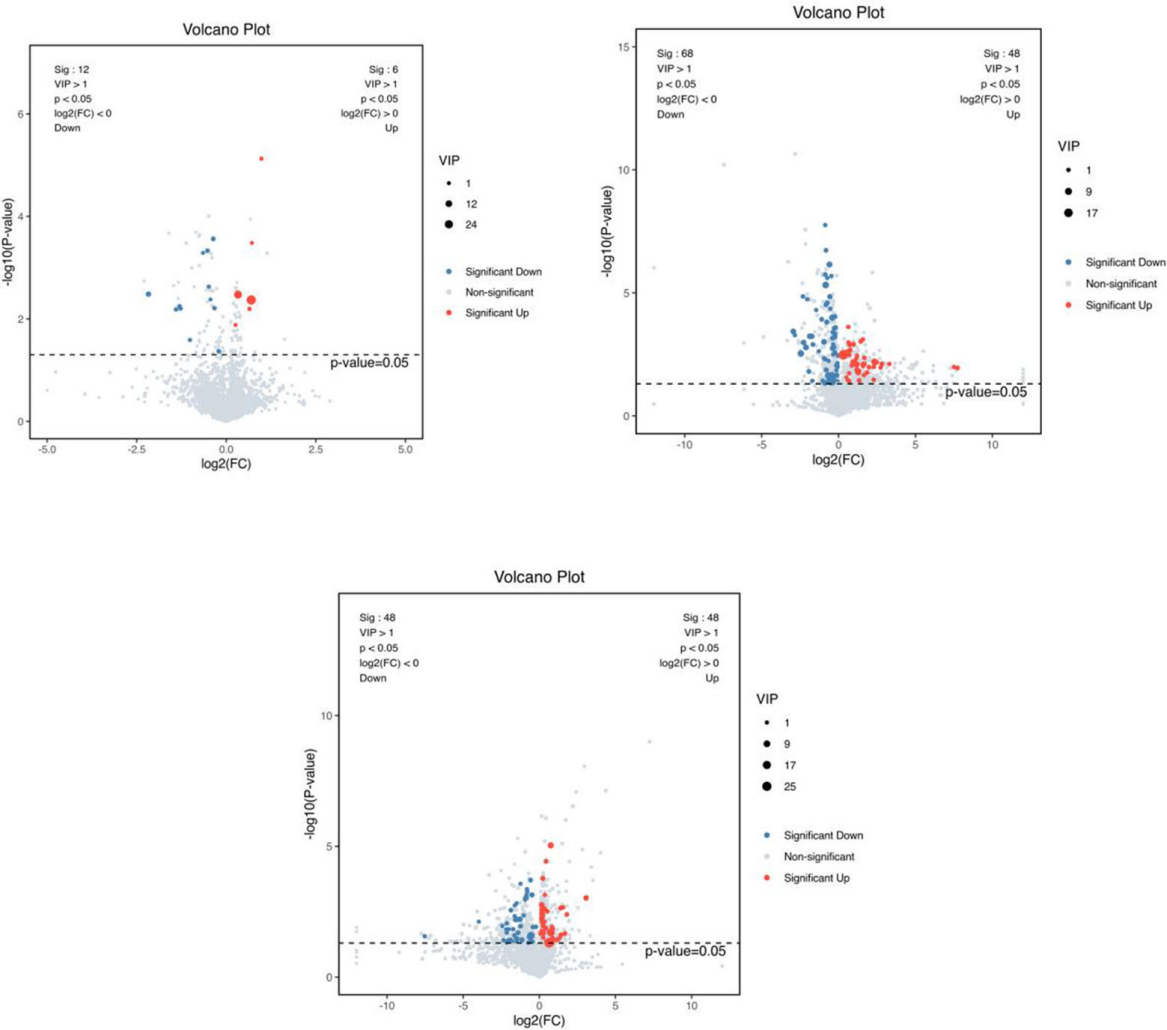
VIP values and screened differential metabolite volcano plots. From left to right, FI group relative to NC group, GSH group relative to NC group and FI group relative to GSH group. The red dots represent differential metabolites that were significantly up-regulated in the experimental groups, the blue dots represent differential metabolites that were significantly down-regulated, and the gray dots represent metabolites that were not significantly different. Each point in the graph represents a metabolite, the horizontal coordinate is the log2 (FC) value of the comparison between the two groups, and the vertical coordinate is the -log10 (p-value) value, with the red dots representing significantly up-regulated differential metabolites (*P*<0.05, VIP>1 and FC>1) and the blue dots representing significantly down-regulated differential metabolites (*P*<0.05, VIP>1 and FC<1).

A combination of multidimensional and unidimensional analyses was used to screen the differential metabolites between groups. The variable weight value (VIP) in Orthogonal Partial Least Squares-Discriminant Analysis is a measure of the expression pattern of each metabolite, the explanatory ability and depth of influence on the categorical discrimination of samples in each group, to explore meaningful differential metabolites, and then use the T-test to verify whether the differential metabolites between groups are significant. As shown in **Figure 5**, a total of 230 differential metabolites were screened in the NC, FI and GSH groups. As can be seen from Volcano **Figure 6**, the FI group up-regulated 6 differential metabolites and down-regulated 12 differential metabolites relative to the NC group; the GSH group up-regulated 48 differential metabolites and down-regulated 68 differential metabolites relative to the NC group; and the FI group up-regulated 48 differential metabolites and down-regulated 48 differential metabolites relative to the GSH group.

#### 3.5.3 Significantly different metabolites

**Figure 7:**
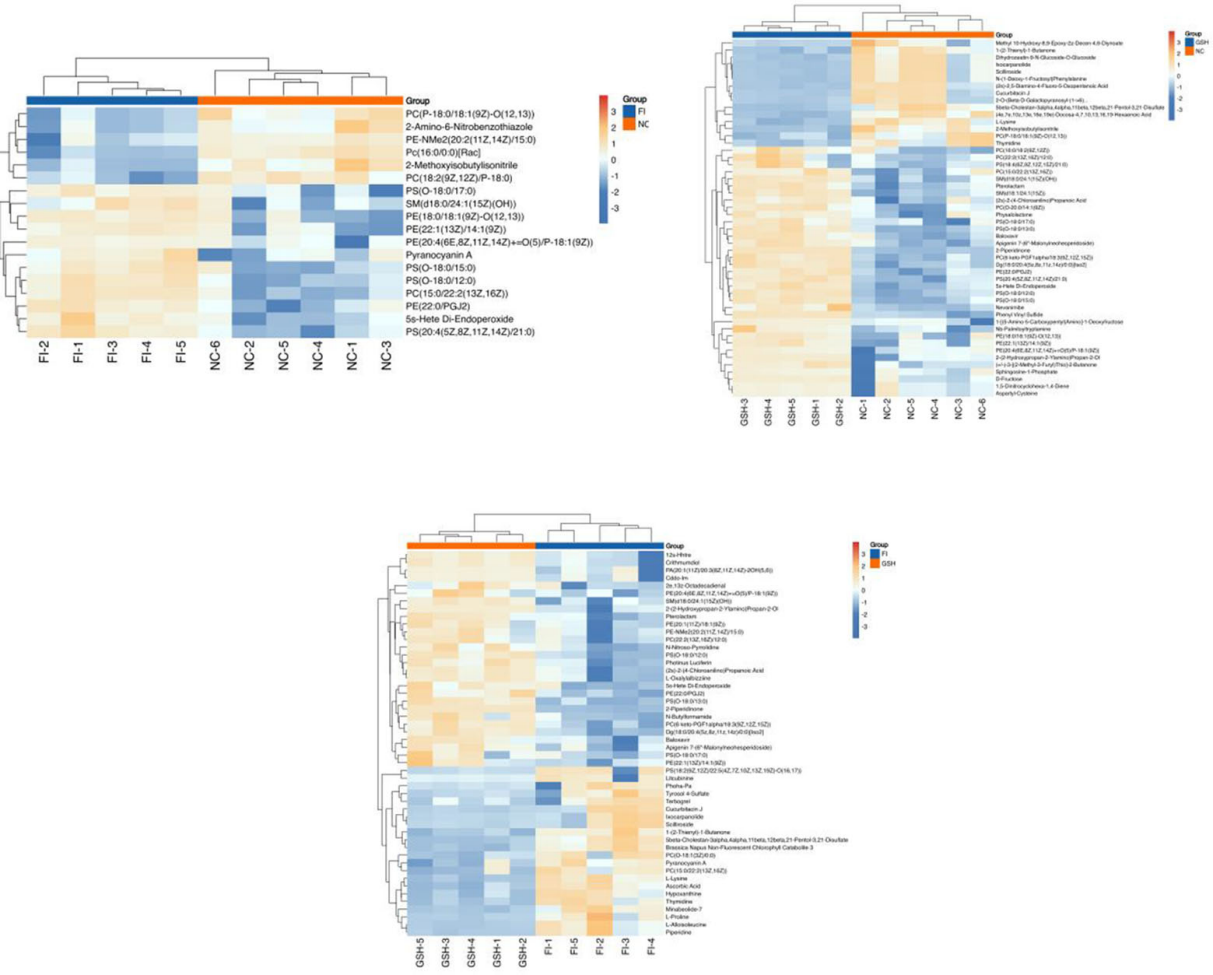
Heatmap of the top 50 differential metabolites of VIP ranking. From left to right, FI group relative to NC group, GSH group relative to NC group and FI group relative to GSH group. Horizontal coordinates indicate sample names and vertical coordinates indicate differential metabolites. The color from blue to red indicates that the expression abundance of the metabolite is from low to high, i.e., the redder it is, the higher the expression abundance of the differential metabolite.

In order to visualize the expression differences of intestinal samples and metabolites among different samples, this study performed hierarchical clustering of the expression levels of all significant differential metabolites among the top 50 significant differential metabolites according to VIP sorting. The results are shown in **Figures 7**. Significant differential metabolites in the three comparisons were PE-NMe2 (20:2(11Z,14Z)/15:0), PC (22:2(13Z,16Z)/12:0), L-Alloisoleucine, PS (O-18:0/13:0), Photinus Luciferin, 5beta-Cholestan-3alpha,4alpha,11beta,12beta,21-Pentol-3,21-Disulfate, Ixocarpanolide, 2-Piperidinone, PS [(18:2(9Z,12Z)/22:5(4Z,7Z,10Z. 13Z,19Z)-O(16,17)], SM [d18:0/24:1(15Z)(OH)], 2e,13z-Octadecadienal, N-Butylformamide, Hypoxanthine, L-Proline, (2s)-2-(4-Chloroanilino) Propanoic Acid, 2-(2-Hydroxypropan-2-Ylamino)Propan-2-Ol.

#### 3.5.4 Metabolic pathway enrichment analysis

**Figure 8:**
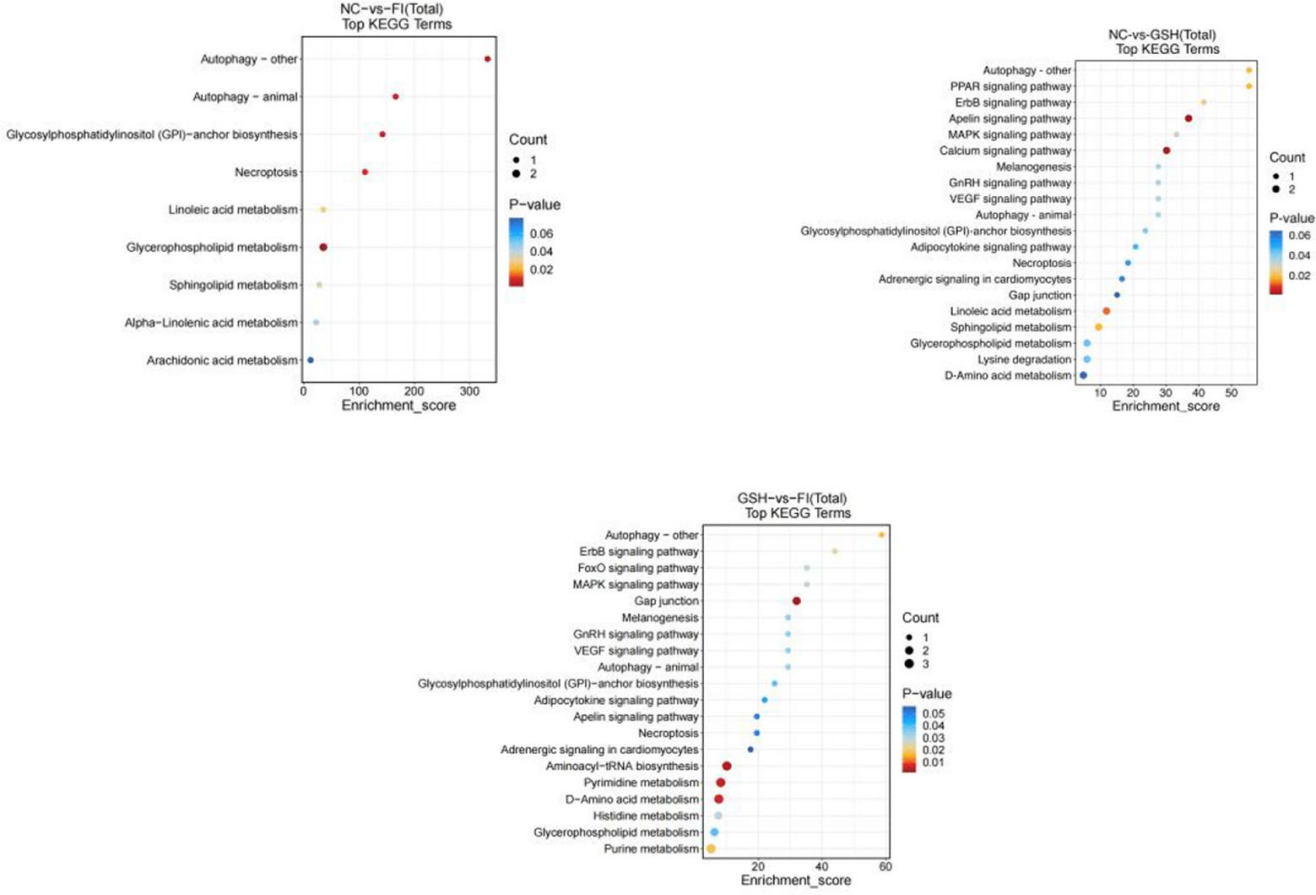
Enrichment bubble diagram of TOP-20 metabolic pathway. From left to right, FI group relative to NC group, GSH group relative to NC group and FI group relative to GSH group. The horizontal coordinate Enrichment Score in the graph is the enrichment score, and the vertical coordinate is the pathway information of top20. The pathways with larger bubbles contain more differential metabolites, the bubble color changes from blue-red, and their enrichment pvalue values are smaller and more significant.

In this study, two groups of intestinal samples were analyzed using the LC-MS metabolomics approach and the differences between the two groups were statistically significant. Based on KEGG metabolic libraries, metabolic pathways with significant differences in metabolites were identified. As shown in **Figures 8**, the major pathways preliminarily analyzed and found to differ between the FI and NC groups were Autophagy-other, Autophagy-animal, Glycosylphosphatidylinositol (GPI) pathway, Calcium signaling pathway, Linoleic acid metabolism, Sphingolipid metabolism; the main pathways differing between the FI group and the GSH group were Gap junction, Aminoacyl-tRNA biosynthesis, Pyrimidine Metabolism, D-Amino acid metabolism. the different pathways shared by the three groups were Autophagy-other, Autophagy-animal, Glycosylphosphatidylinositol (GPI)-anchor biosynthesis, Necroptosis, Glycerophospholipid metabolism.

### 3.6 Modulation of hepatic lipid metabolites in bullfrogs by glutathione mix feeding

#### 3.6.1 Sample raw data and principal component analysis

**Figure 9:**
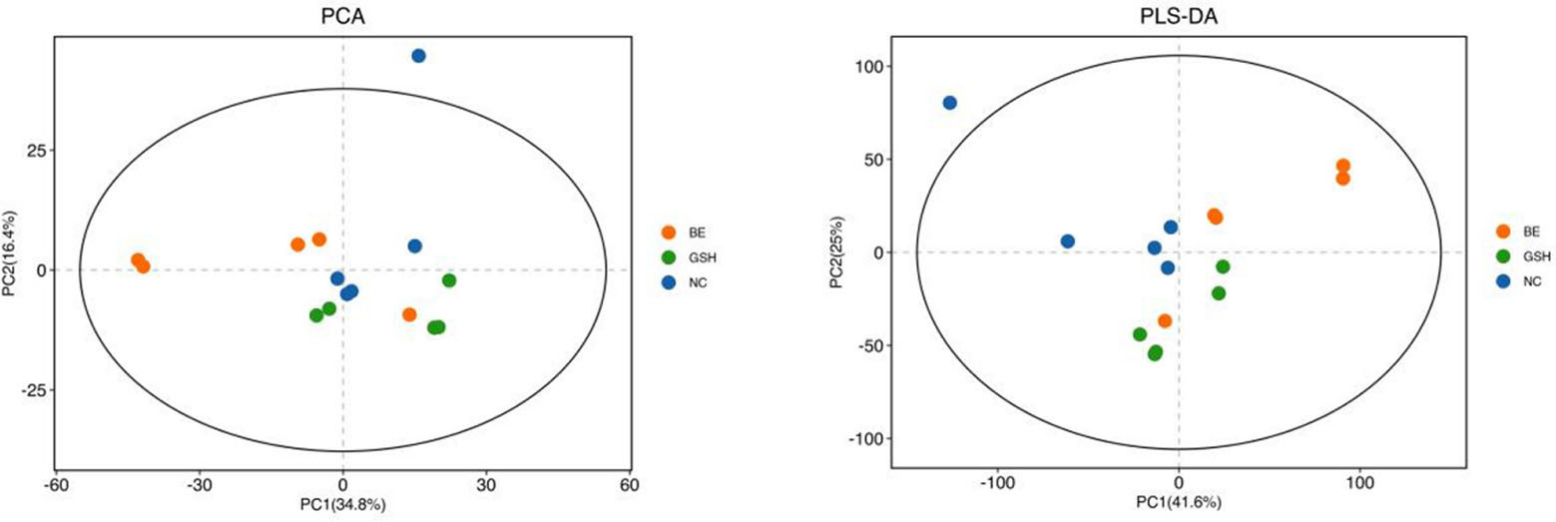
PCA vs. PLS-DA plots of hepatic lipid metabolites in the NC, FI, and GSH groups.

As can be seen from **Figure 9**, the samples of NC, FI and GSH groups were all in the 95% confidence interval, and could be spatially separated evenly without overlap, indicating that there were obvious differences in metabolites among the three groups, and they were able to be analyzed in the next step.

#### 3.6.2 Screening of differential metabolites

**Figure 10:**
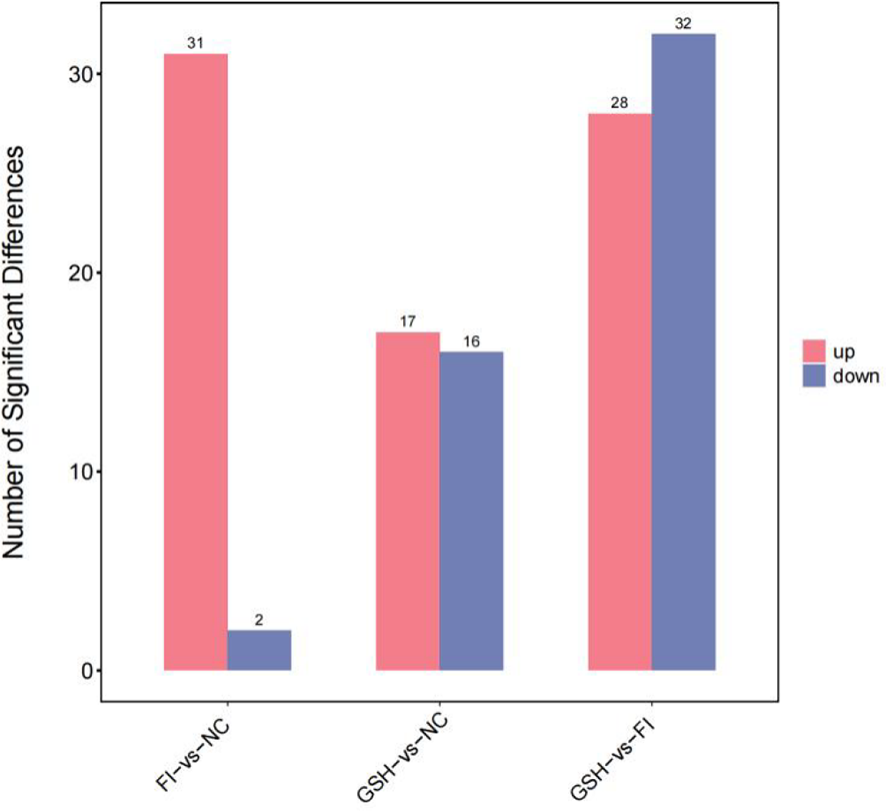
Differential metabolite quantity statistical chart.

**Figure 11:**
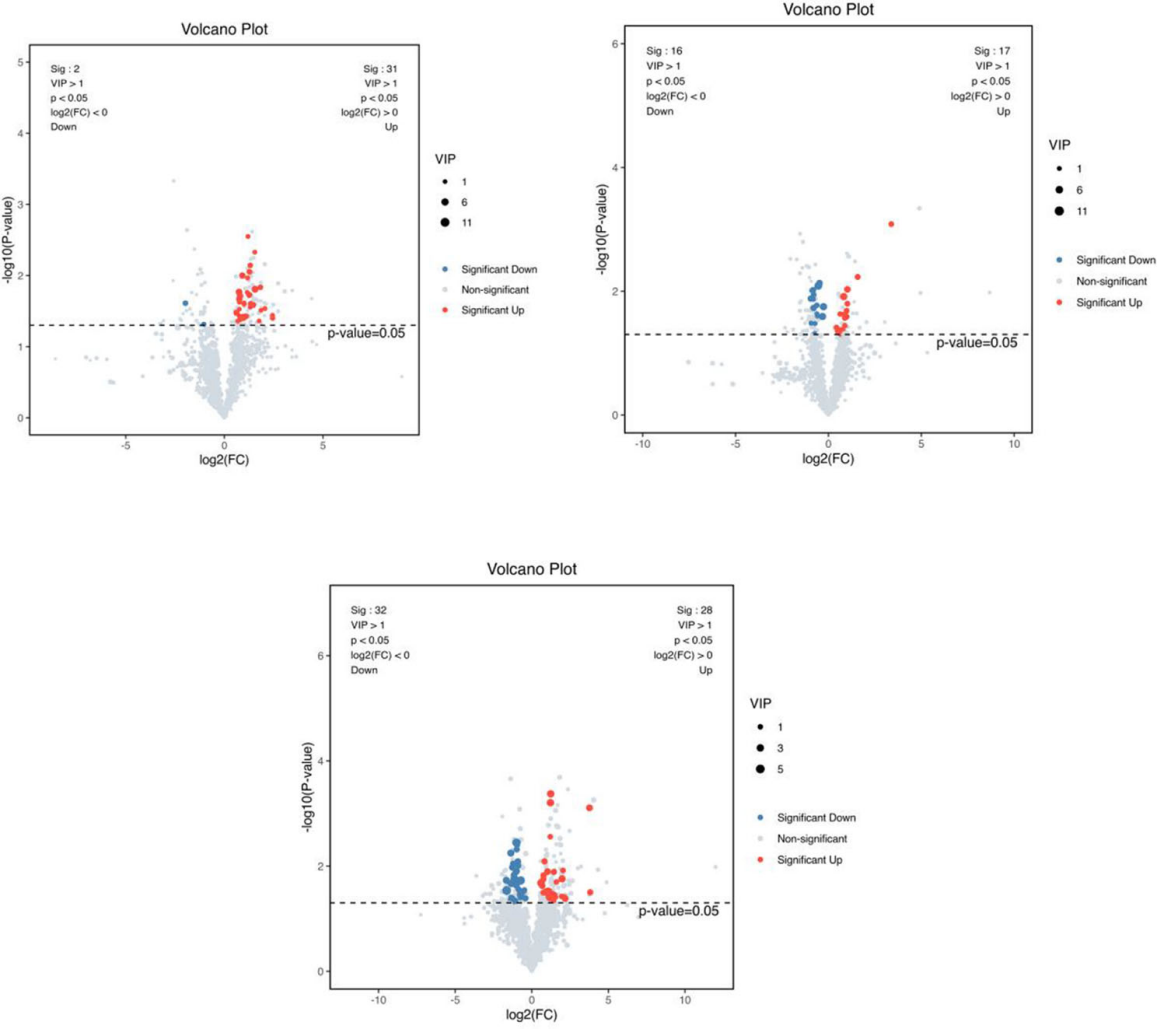
VIP values and screened differential metabolite volcano plots. From left to right, FI group relative to NC group, GSH group relative to NC group and FI group relative to GSH group. The red dots represent differential metabolites that were significantly up-regulated in the experimental groups, the blue dots represent differential metabolites that were significantly down-regulated, and the gray dots represent metabolites that were not significantly different. Each point in the graph represents a metabolite, the horizontal coordinate is the log2 (FC) value of the comparison between the two groups, and the vertical coordinate is the -log10 (p-value) value, with the red dots representing significantly up-regulated differential metabolites (*P*<0.05, VIP>1 and FC>1) and the blue dots representing significantly down-regulated differential metabolites (*P*<0.05, VIP>1 and FC<1).

As shown in **Figure 10**, a total of 230 differential metabolites were screened in the NC, FI and GSH groups. As can be seen from Volcano **Figure 11**, the FI group up-regulated 31 differential metabolites and down-regulated 2 differential metabolites relative to the NC group; the GSH group up-regulated 17 differential metabolites and down-regulated 16 differential metabolites relative to the NC group; and the FI group up-regulated 28 differential metabolites and down-regulated 32 differential metabolites relative to the GSH group.

#### 3.6.3 Significant differential metabolites

**Figure 12:**
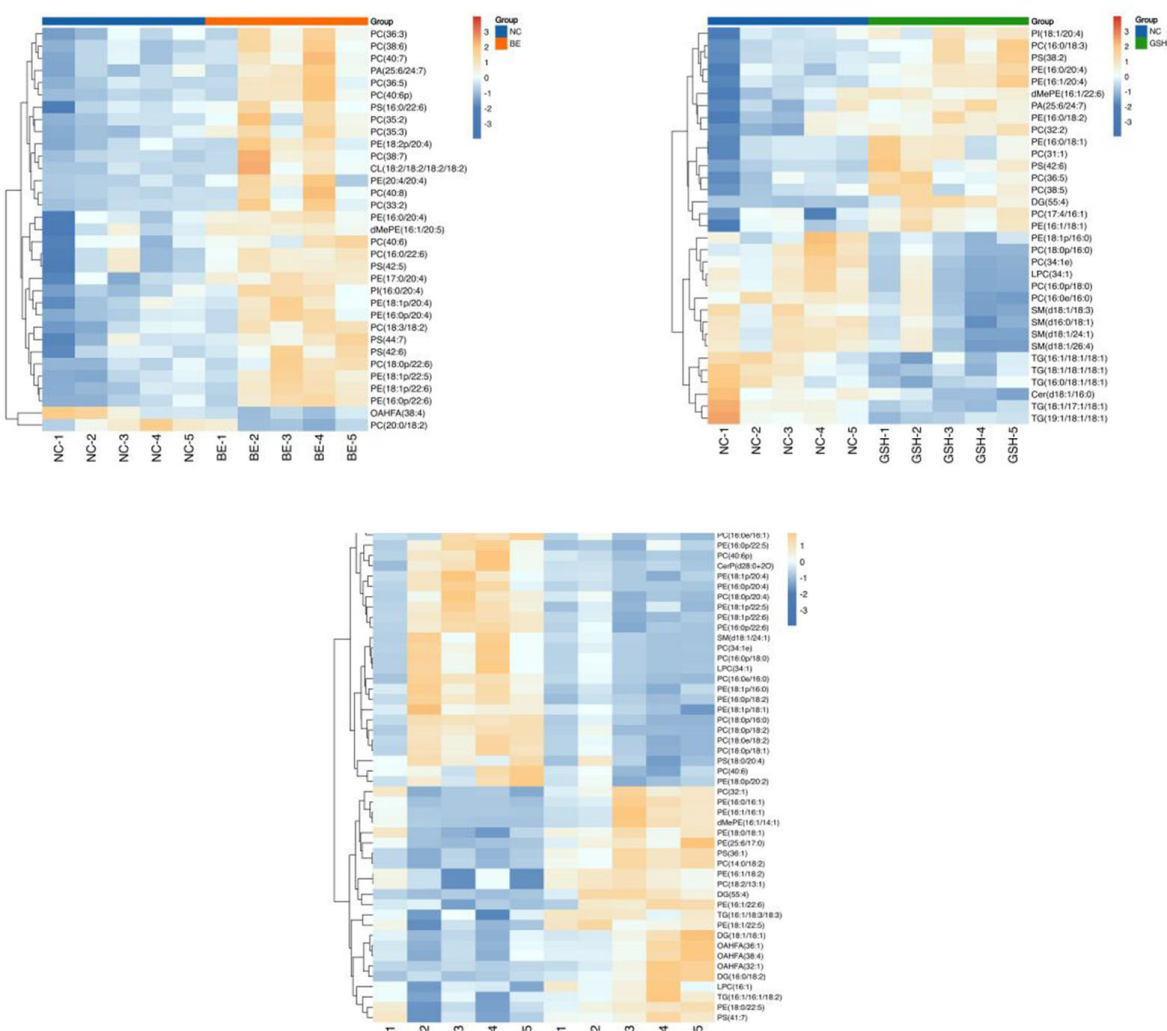
Heatmap of the top 50 differential metabolites of VIP ranking. From left to right, FI group relative to NC group, GSH group relative to NC group and FI group relative to GSH group. Horizontal coordinates indicate sample names and vertical coordinates indicate differential metabolites. The color from blue to red indicates that the expression abundance of the metabolite is from low to high, i.e., the redder it is, the higher the expression abundance of the differential metabolite.

The results are shown in **Figure 12**. The significant differential metabolites in the three group comparison were PC (36:3), PC (38:6), PE (16:0/20:4), PC (16:0/22:6), PS (42:5), PI (16:0/20:4), PE (18:1p/20:4), dMePE (16:1/20:5), PE (16:0p/20:4), PC (36:5), PE (18:1p/22:5), OAHFA (38:4), PC (40:7), PE (18:2p/20:4), PC (40:6p).

#### 3.6.4 Metabolic pathway enrichment analysis

**Figure 13:**
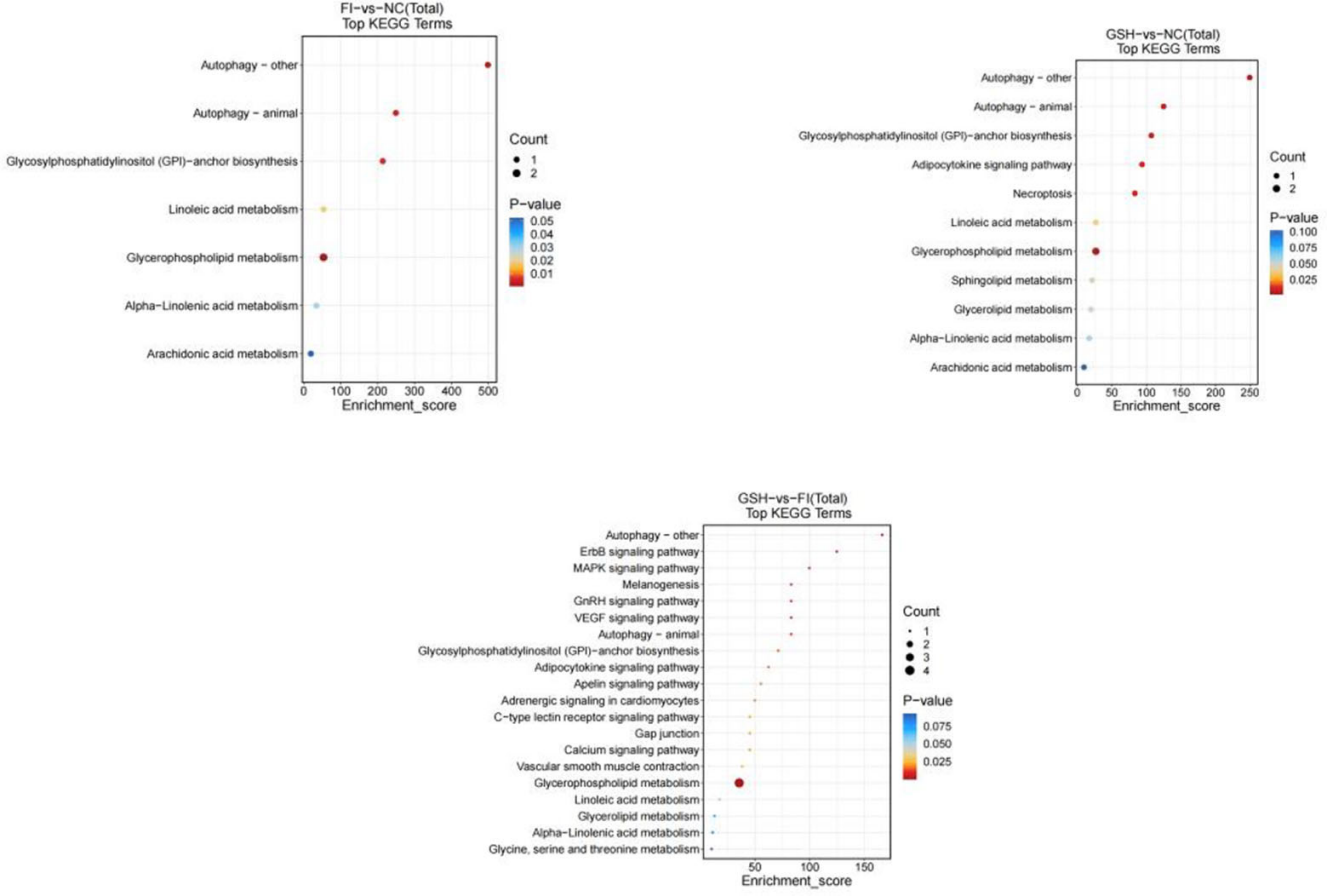
Enrichment bubble diagram of TOP-20 metabolic pathway. From left to right, FI group relative to NC group, GSH group relative to NC group and FI group relative to GSH group. The horizontal coordinate Enrichment Score in the graph is the enrichment score, and the vertical coordinate is the pathway information of top20. The pathways with larger bubbles contain more differential metabolites, the bubble color changes from blue-red, and their enrichment pvalue values are smaller and more significant.

As shown in **Figure 13**, preliminary analysis and found that the main difference pathways between FI group and NC group were Autophagy-other, Autophagy-animal, Glycosylphosphatidyylinositol (GPI)-anchor biosynthesis, Glycerophospholipid metabolism; the main pathways of difference between the GSH group and the NC group were Autophagy-animal, Glycosyl phosphatidylinositol (GPI)-anchor biosynthesis, Linoleic acid metabolism; FI group and the NC group were mainly differentiated by the following pathways Glycosylphosphatidylinositol (GPI)-anchor biosynthesis, Linoleic acid metabolism; the main pathways differing between the FI group and the GSH group were Autophagy-other, ErbB signaling pathway, MAPK signaling pathway, and glycerophospholipids metabolism; and the pathways differing between the FI group and the GSH group were Autophagy-other, Autophagy-animal, and Autophagy-animal, Glycosylphosphatidylinositol (GPI)-anchor biosynthesis, Glycerophospholipid metabolism.

## 4 Discussion

### 4.1 Effect of glutathione on growth performance and hepatic lipid metabolism in bullfrogs

Relatively many studies have been conducted to show that glutathione has a promoting effect on the growth and antioxidant capacity of aquatic animals (Kwon et al., 2024). Zhao et al. (Hongxia Zhao et al., 2008) found that the addition of a certain amount of GSH to the diet could significantly improve the growth performance of grass carp at the initial stage and promote the nutritional metabolism of the fish, and it was hypothesized that GSH might increase the activity of growth hormone by destroying growth inhibitors, and at the same time, it promoted lipolysis and enhanced the oxidation of fatty acids, thus raising the cholesterol level, but the high dosage of GSH might cause some damage to the liver of grass carp. Zhou (Zhou, 2018) found that the weight gain rate of Pelteobagrus fulvidraco increased with the increase of GSH in the diet, and they were all significantly higher than that of the control group, and the cholesterol and triglycerides of the 100–500 mg/kg group were higher than that of the control group, which indicated that the ability of lipid metabolism was enhanced. In this experiment, with the use of glutathione, the survival rate of bullfrog culture was improved, which inferred that glutathione can effectively regulate the liver lipid metabolism of bullfrogs and improve the growth function of bullfrogs. Yi et al. (Jianhua Yi et al., 2023) also found that the addition of 350 mg/kg of GSH to the feed significantly increased the final body mass, weight gain rate and specific growth rate of cultured Pelteobagrus fulvidraco, while significantly reducing the feed coefficient, and at the same time, the HDL cholesterol in the blood was elevated. Feng et al. (Feng Ju Pan et al., 2021) showed that the addition of GSH at a level greater than 0.18 g/kg significantly increased the growth rate and reduced the bait coefficient of crayfish. It was hypothesized that it was related to its anti-stress effect, but further research and study from the perspective of material energy metabolism is needed (Jang et al., 2024). Liu (Liu, 2020) found that the addition of appropriate amount of GSH to the diet could improve the stress resistance and growth performance of cultured Chinese mitten crab. Cao et al. (Junming Cao et al., 2008) found that the addition of GSH to the diet could increase the GSH content in the hepatopancreas of the shrimp Penaeus vannamei, which was hypothesized to affect the growth of the shrimp by influencing a series of physiological activities of the shrimp, and improve the feed conversion efficiency and survival rate. The results of liver tissue sections in the experiment showed that the liver tissue of bullfrogs in the GSH group was repaired to a certain extent relative to that of the FI group after the use of glutathione mixed with bullfrog feed, indicating that glutathione has a better therapeutic effect on the liver of bullfrogs.

### 4.2 Serum antioxidant capacity and effect on hepatic lipid metabolizing capacity in bullfrogs with glutathione

The animal body due to the normal metabolic process is prone to produce many less stable unpaired electron molecules, atomic groups or chemical groups (Liu et al., 2024a), known as free radicals, and superoxide anion radicals (O2-) is one of the main forms of free radicals (Abdelazim and Abomughaid, 2024). However, O2- can oxidize phospholipids on the cell membrane to produce peroxides such as malondialdehyde (MDA) and ketone groups containing aldehyde groups to destroy the cell membrane structure (Tao et al., 2024), thus causing a series of diseases (He et al., 2024a). Antioxidant enzymes such as superoxide dismutase (SOD) and catalase (CAT) are the most important defense system to remove O2- in the body, reflecting the vitality of antioxidant enzymes in the body (Medinas and Augusto, 2010). Among them, SOD is an important antioxidant enzyme class that exclusively defends against excessive O2- during metabolic processes in the organism, and its activity level indirectly reflects the degree of oxidative damage suffered by the organism (Chen et al., 2023). In this experiment, the use of glutathione mixed with bullfrog feed accelerated the normal metabolic rate in the body of bullfrogs, which produced excess O2-. In order to scavenge excess O2- and its peroxidized products such as MDA, the activity of SOD enzyme in the GSH group was significantly higher than that in the FI group, which indicated that glutathione could effectively improve the antioxidant capacity of bullfrogs. Alkaline phosphatase (AKP) is present in many tissues (Liu et al., 2019), but the highest concentrations are found in the liver, biliary epithelium and bone (Chen et al., 2003). Detection of this enzyme is important in determining liver disease. Serum acid phosphatase (ACP) is mainly derived from the liver, erythrocytes and platelets In hepatitis, cirrhosis, abnormal liver metabolism, and extrahepatic and intrahepatic obstructive biliary diseases and cirrhosis (Peng et al., 2015), the enzyme levels of AKP and ACP are greatly increased (Shang et al., 2003). In this experiment, glutathione feeding significantly reduced the enzyme levels of AKP and ACP, and effectively alleviated the symptoms of abnormal liver lipid metabolism and hepatitis in bullfrogs.

Total cholesterol, including free cholesterol and cholesteryl esters, is synthesized and stored in the liver, and its serum concentration can be used as an indicator of lipid metabolism (Cai et al., 2024b). High density lipoprotein cholesterol (HDL-C), mainly synthesized in the liver, is an anti-atherosclerotic lipoprotein that transports cholesterol from extrahepatic tissues to the liver for metabolism, and it is excreted from the body by bile (Deng et al., 2024). Lower HDL cholesterol is commonly seen in cerebrovascular disease coronary heart disease, hypertriglyceridemia, liver function damage such as acute and chronic hepatitis, cirrhosis, and liver lipid metabolism abnormalities (Quetglas-Llabres et al., 2024). The activities of HDL-C and T-CHO in the FI group were significantly lower than those in the NC group in this experiment; while the activities of LDL-C, TBA and TG were significantly higher. Studies have shown that abnormal liver metabolism and impaired hepatic synthesis of LDL can cause low LDL (Wu et al., 2024a). In contrast, increased total bile acids are commonly found in various acute and chronic hepatitis, alcoholic hepatitis, as well as in the vast majority of extrahepatic bile duct obstruction and intrahepatic cholestatic diseases, cirrhosis, and so on. In living organisms, the synthesis and catabolism of triglycerides are key aspects of energy metabolism (Zhang et al., 2023; Mireault et al., 2024). They not only serve as energy storage and provision substances, but also participate in the construction of cellular structures and regulate the properties of biological membranes (Hu and Du, 2023). In the field of medicine, triglyceride levels are one of the most important indicators of the health of a living organism. Increased serum triglycerides can be seen in hypothyroidism, nephrotic syndrome, pancreatitis, impaired liver function and abnormal lipid metabolism (Chen et al., 2024; Guo et al., 2024b). And with the use of glutathione, the activities of LDL-C, TBA and TG of bullfrogs were reduced, which proved that glutathione has a better therapeutic effect on the abnormalities of hepatic lipid metabolism in bullfrogs.

### 4.3 Regulation of serum metabolites and hepatic metabolites in bullfrogs by glutathione

In the serum metabolomics and liver metabolite assay of bullfrogs, it was found that the significant differential metabolites in the three comparisons were mainly lipids and lipid-like molecules, as well as some organic acids and their derivatives. And these lipids and lipid-like molecules were dominated by glycerophospholipids. Glycerophospholipids are the most common phospholipids (Liu et al., 2024b). In glycerophospholipids, two of the hydroxyl groups of glycerol form esters with fatty acids, and the third hydroxyl group is esterified by phosphoric acid, the product being phosphatidic acid (Wu et al., 2024b). Phosphatidic acid is a polar lipid because the bound phosphate has a dissociable carboxyl group. Glycerophospholipids are involved in a variety of neural and intracellular signaling and are important components of cell membranes. In addition, it is involved in the synthesis of bile, cell signaling, and the maintenance of normal metabolism in the body (Kim et al., 2024; Wang et al., 2024c). Glycerophospholipids are derivatives of 3-phosphoglycerol, including phosphatidylcholine, phosphatidylserine, phosphatidylethanolamine, and phosphat- idylinositol, among others (Shi et al., 2024). Relevant studies have shown that the four glycerophospholipids, as one of the main components of the cell membrane, can maintain the integrity and stability of the cell membrane, preventing the rupture and lysis of the cell membrane. PE is able to establish hydrogen bonding with a variety of amino acid residues, thereby inhibiting the localization of negative amino acids on the cytoplasmic side, and it is an important determinant of the topology of transmembrane domains of membrane proteins (Wang et al., 2024a). PC is essential for the normal secretion of very low density lipoproteins (VLDL) by hepatocytes, and it is also essential for the secretion of various types of lipoproteins (Ibrahim et al., 2024). PC is required for the normal secretion of very low density lipoprotein (VLDL) from hepatocytes and is also a major source of various second messengers, which are involved in cellular signaling (Shatursky et al., 2024). PS can regulate cellular signaling by interacting with other proteins, thereby affecting cell proliferation and differentiation. In addition, PS can participate in the coagulation process, regulating platelet aggregation and vasoconstriction (Naozuka et al., 2024). And PE is able to establish hydrogen bonds with a variety of amino acid residues, which in turn inhibits the localization of negative amino acids on the cytoplasmic side, and is an important determinant of the topology of transmembrane structural domains of membrane proteins (Shi et al., 2024). The results of the serum metabolome in this experiment showed that the levels of PC, PS, and PE were significantly decreased in the FI group, whereas their levels were significantly up-regulated after the intervention of glutathione administration, suggesting that glutathione alleviates glycerophospholipid metabolism disorders to a certain extent, and thus hepatic lipid metabolism disorders.

With the use of glutathione, the Autophagy - other and ErbB signaling pathways are up-regulated and the Sphingolipid metabolism pathway is down-regulated.Sauer K et al. (Sauer et al., 2022) found that autophagy is a catabolic “self-feeding” pathway. Sauer K et al. found that autophagy is a catabolic “self-feeding” pathway that is emerging as a key integration point in cellular physiology (Wang et al., 2024b). Using its own set of genes, the autophagy pathway communicates with virtually all signaling networks and organelles. Whereas autophagy involves synthesizing double membrane autophagosomes from scratch, sequestering selected cellular contents, and then delivering the sequestered contents to vesicles (yeast and plants) or lysosomes (animal cells) for degradation and recirculation, it was also found in the studies of Komatsu M (Komatsu et al., 2005) that this process can be either constitutive or adaptive. The first form controls the energy balance of cells and tissues by eliminating senescent or damaged organelles. The latter form involves the mobilization of intracellular energy reserves in response to nutritional stress or deficiency. In mammals, the first metabolic function induced by autophagy is the release of amino acids through protein degradation during starvation. The released amino acids not only maintain protein synthesis under fasting conditions but also provide food for the tricarboxylic acid cycle and promote ATP production. In addition Ezaki J (Ezaki et al., 2011) showed that hepatic autophagic protein hydrolysis releases amino acids via gluconeogenesis to produce glucose, which significantly maintains blood glucose during fasting. Studies by Natasha C (Lucki and Sewer, 2012) have shown that nuclear lipid metabolism is involved in a wide variety of processes including transcription, splicing and DNA repair. Sphingolipids play a role in many cellular functions, and the emerging literature has identified roles for these lipid mediators in different nuclear processes. Different sphingolipid species are localized in different subnuclear structural domains, including chromatin, nuclear matrix, and nuclear envelope, where sphingolipids perform specific regulatory and structural functions. sphingomyelin is the most abundant nuclear sphingolipid, and in addition to being an integral part of the nuclear matrix, it also plays structural and regulatory roles in chromatin assembly and dynamics (Yuan et al., 2024).

Amino acids affect the synthesis of lipids, glutathione, nucleotides, etc. The liver plays an integral role in maintaining amino acid balance (Liu et al., 2024b). It has been found that when the liver is damaged, hormones that promote amino acid catabolism increase or decrease accordingly, resulting in increased or decreased levels of amino acid catabolism and amino acids in the systemic circulation (He et al., 2024b). Amino acid related metabolic pathways were disrupted in both serum metabolic group and liver metabolic group in this experiment. Necroptosis metabolic pathway was significantly down-regulated in FI group comparing to NC group. Onal et al. (Onal et al., 2023) found that mixed-spectrum kinase structural domain-like pseudokinase (MLKL) is an indispensable end mediator of necroptosis (Cheng et al., 2024). The study was performed in the serum metabolic group and liver metabolic group of this experiment. Necroptosis, also known as programmed cellular necrosis, is a cysteine asparaginase-independent cell death mechanism involved in a variety of pathological and inflammatory processes. Necroptosis is regulated at multiple levels, from the stability and post-translational modification of transcriptional to necroptotic components, to the availability of molecular interacting partners and receptor-interacting serine/threonine protein kinase 1 (RIPK1), receptor interacting serine/threonine protein kinase 3 (RIPK3) and mixed spectrum kinase structural domain-like protein (MLKL) (Ma et al., 2024). In contrast, the GSH group significantly down-regulated the D-Amino acid metabolism pathway in comparison to the FI group. ruby Guo et al. (Guo et al., 2024a) showed that an approach utilizing yeast d-amino acid oxidase (DAAO) was able to manipulate intracellular redox metabolism through the production of hydrogen peroxide in the presence of d-amino acids and led to the development of new and informative animal models to characterize the effects of oxidative stress on heart failure and neurodegenerative diseases (Guo et al., 2024a). Amin et al. (Nanji et al., 2001) found that arginine not only reduces endotoxin and lipid peroxidation in mice with alcohol-induced liver injury, but also reduces the levels of inflammatory factors. Arginine is formed from proline catalyzed by arginine synthase and can be oxidized by nitric oxide synthase to citrulline, which is then hydrolyzed to ornithine. Related studies have shown that ornithine activates ornithine carbamoyltransferase and carbamoylphosphate synthetase, key enzymes in the process of urea synthesis, and binds to circulating ammonia to accelerate urea synthesis and ammonia metabolism, thereby restoring hepatocyte function. Therefore, it is hypothesized that glutathione interferes with the synthesis and catabolism of the differential metabolite arginine through significant down-regulation of the D-Amino acid metabolism pathway and up-regulation of the Necroptosis metabolism pathway, thus further affecting the production and metabolism of urea to exert a protective effect against liver injury in bullfrogs.

## 5 Conclusions

Summarizing the above results, we speculate that glutathione can protect the liver of bullfrogs by repairing liver tissues, enhancing the antioxidant ability of bullfrogs, affecting the related metabolism of the body, repairing liver damage, up-regulating Autophagy - other, Necroptosis and ErbB signaling pathway, and It also up-regulated Autophagy - other, Necroptosis and ErbB signaling pathway, and down-regulated Sphingolipid metabolism and D-Amino acid metabolism pathway, and regulating the disorder of liver lipid metabolism. The final performance of the bullfrog liver lipid metabolism disorder was alleviated, promoting the normal growth of bullfrogs and improving the survival rate of bullfrog farming. In conclusion, our results concluded that glutathione is capable of effectively alleviating hepatic lipid metabolism disorders and treating hepatitis, with a view to providing a reference for future in-depth research on the mechanism of action of glutathione, promoting healthy and green culture of bullfrogs, and being of great significance for bullfrog food safety and ecological safety.

## Data availability statement

The original contributions presented in the study are included in the article/supplementary material. Further inquiries can be directed to the corresponding author/s.

## Ethics statement

The animal study was approved by Shanghai Ocean University Animal Ethics Committee. The study was conducted in accordance with the local legislation and institutional requirements.

## Author contributions

ZS: Writing – original draft, Writing – review & editing, Data curation, Investigation, Methodology, Project administration, Validation. FG: Conceptualization, Writing – review & editing. RS: Methodology, Writing – review & editing. KC: Formal analysis, Writing – review & editing. SF: Investigation, Writing – review & editing. XL: Writing – review & editing. DL: Writing – review & editing. KH: Writing – review & editing.
